# Protective Effect of Resolvin D1, D2, and Their Methyl Esters on Oxidative Stress and Hyaluronidase—Induced Hyaluronic Acid Degradation

**DOI:** 10.3390/antiox15020163

**Published:** 2026-01-25

**Authors:** Zahra Kariminezhad, Mahdi Rahimi, Julio Fernandes, Hassan Fahmi, Mohamed Benderdour

**Affiliations:** 1 Orthopedic Research Laboratory, Hôpital du Sacré-Cœur de Montreal, Université de Montreal, Montreal, QC H4J 1C5, Canada; zahra.kariminezhad.cnmtl@ssss.gouv.qc.ca (Z.K.); mahdi.rahimi.hoseinabad.cnmtl@ssss.gouv.qc.ca (M.R.); julio.c.fernandes@umontreal.ca (J.F.); 2 Osteoarthritis Research Unit, University of Montreal Hospital Research Center (CRCHUM), Montreal, QC H2X 0A9, Canada; h.fahmi@umontreal.ca

**Keywords:** viscosupplementation, osteoarthritis, hyaluronic acid, Resolvin derivatives, antioxidants, enzyme inhibition, hyaluronidase

## Abstract

Hyaluronic acid (HA) injections are commonly employed in the management of osteoarthritis (OA), yet their therapeutic benefits are often limited by oxidative degradation and enzymatic breakdown within the joint. This study investigates whether Resolvin D1, Resolvin D2, and their methyl ester derivatives can enhance the efficacy of HA injections by acting as dual-function agents with both antioxidant and enzyme inhibitory properties. A comprehensive series of in vitro assays—including ORAC, FRAP, DPPH, ABTS, HRS, and SOD—were performed to evaluate antioxidant capacity, using Trolox, Ascorbic acid, β-Carotene, and Quercetin as reference standards. The potential to inhibit HA degradation was assessed through ROS-induced HA fragmentation and hyaluronidase inhibition assay, with epigallocatechin gallate (EGCG) serving as a positive control. The results indicate that Resolvin derivatives, particularly the methyl ester form of Resolvin D1, display mechanism-dependent antioxidant activity, showing pronounced effects in hydrogen atom transfer-based assays (e.g., ORAC and HRS), as well as in ABTS^•+^ and superoxide-related systems, along with protection against ROS and enzyme-induced HA degradation. These findings suggest that incorporating Resolvin derivatives may represent a promising strategy to improve HA-based viscosupplementation by enhancing stability and therapeutic persistence in osteoarthritic joints.

## 1. Introduction

**Figure 1:**
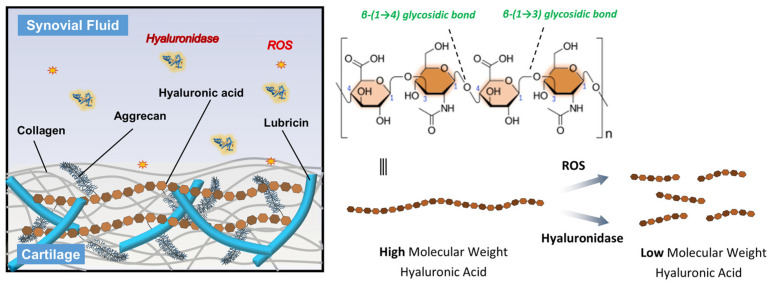
Schematic representation of the HA structure and its degradation mechanisms. HA consists of repeating disaccharide units of N-acetyl-D-glucosamine and D-glucuronic acid linked by alternating β-(1 → 4) and β-(1 → 3) glycosidic bonds. In OA joints, high molecular weight HA is degraded into low molecular weight fragments through the action of ROS and hyaluronidase enzymes, leading to reduced viscosity and diminished therapeutic efficacy.

Osteoarthritis (OA) is characterized by the progressive degeneration of articular cartilage, inflammation of the synovial membrane, and a decline in joint function, often resulting in pain, stiffness, and reduced mobility [1]. One of key component in maintaining joint health is hyaluronic acid (HA), a naturally occurring glycosaminoglycan composed of repeating disaccharide units of N-acetylglucosamine and glucuronic acid (Figure 1) [2,3]. HA plays a crucial role in maintaining joint health, primarily due to its high molecular weight (average Mw in human synovial ≈ 7 million Dalton), which contributes to the viscoelastic properties of synovial fluid [4]. In the context of OA and other joint diseases, HA provides lubrication, shock absorption, and supports cartilage integrity [5]. However, in diseased joints, the concentration and molecular weight of HA often decrease, compromising its function due to the degradation [6]. The degradation is accelerated by the enzymes, immune cells, and oxidant substances, which breaks down HA into smaller fragments, reducing its ability to protect the joint [6]. Excessive HA degradation contributes to inflammation, pain, and cartilage deterioration. For instance, a newly discovered enzyme, HYBID, which degrades hyaluronan, is found in various human cells. Importantly, HYBID has been observed at elevated levels in osteoarthritic chondrocytes and fibroblast-like synoviocytes [7]. This suggests a strong connection between increased HYBID and the deterioration of cartilage and HA in the joints of individuals with OA.

Furthermore, the overproduction of reactive oxygen species (ROS) plays a very crucial role in the progression of joint degeneration [8]. ROS, including hydrogen peroxide (H_2_O_2_), hydroxy radical (OH^−^), and superoxide anion (O_2_^−^) are generated by activated chondrocytes and synoviocytes in response to mechanical stress and inflammatory cytokines [9]. These reactive molecules can directly damage joint tissues by inducing oxidative stress, leading to degradation of extracellular matrix components such as collagen and proteoglycans [10]. Importantly, ROS also contribute to the depolymerization and degradation of HA in the synovial fluid, reducing its molecular weight and impairing its viscoelastic and lubricating properties (Figure 1) [11,12]. This loss of HA function exacerbates joint inflammation and cartilage breakdown, further accelerating OA progression. Therefore, protecting HA from degradation is essential for maintaining joint homeostasis and managing OA symptoms.

**Figure 2:**
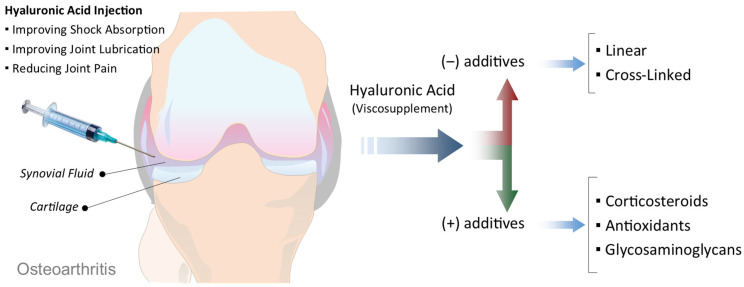
Schematic illustration of viscosupplementation in OA. Intra-articular injection of HA into the OA joint aims to improve shock absorption, enhance joint lubrication, and reduce pain. HA-based viscosupplements vary in their formulation, ranging from linear to cross-linked structures (without additives), or combined with additional components such as corticosteroids, antioxidants, or glycosaminoglycans to enhance therapeutic effects.

Viscosupplementation is a minimally invasive treatment option for OA, most commonly used for the knee joint, although it may also be applied to other joints such as the hip, ankle, or shoulder [13,14]. The procedure involves the intra-articular injection of HA that plays a critical role in maintaining the viscosity, elasticity, and lubricating properties of synovial fluid [15]. In OA joints, both the quantity and quality of endogenous HA are significantly reduced, leading to increased friction, cartilage wear, inflammation, and pain [16,17]. The primary goal of viscosupplementation is to restore the viscoelasticity of the synovial fluid, thereby improving shock absorption, joint lubrication, and overall function (Figure 2) [18,19]. Clinically, this often results in reduced joint pain, increased mobility, and, in some cases, a delay in the progression of the disease or postponement of joint replacement surgery. Several FDA-approved HA formulations are currently available for viscosupplementation, differing in aspects such as molecular weight, origin (e.g., avian-derived vs. biofermentation-based), cross-linking, and dosage regimen (single injection vs. multiple weekly injections). Commonly used products include Hyalgan, Synvisc and Synvisc-One, Supartz, Orthovisc, and Durolane [16,20]. Some formulations, such as Cingal, combine HA with a corticosteroid to provide both long-term joint lubrication and immediate anti-inflammatory relief, offering a potentially cost-effective and efficient solution for symptomatic management [21,22].

**Figure 3:**
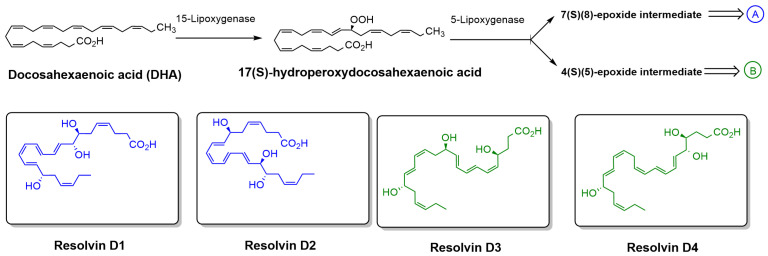
Biosynthetic pathway of RvDs derived from DHA. DHA is enzymatically converted by 15-LOX to 17(S)-hydroperoxydocosahexaenoic acid, which is then further metabolized by 5-LOX to form either a 7(S),(8)-epoxide intermediate (pathway A) or a 4(S),(5)-epoxide intermediate (pathway B). These intermediates lead to the formation of specific RvDs, including Resolvin D1 and D2 (pathway A) and Resolvin D3 and D4 (pathway B). The chemical structures of Resolvin D1–D4 are also shown.

There are several synthetic antioxidants widely used in the food and pharmaceutical industries to prevent oxidative degradation; however, recent findings have raised safety concerns regarding their potential to inhibit vital enzymes [23]. As a result, there is a growing demand for safer, biodegradable alternatives with fewer health risks. Among the various natural compounds under investigation, lipid-derived molecules with anti-inflammatory and antioxidative properties have attracted considerable attention. One such promising group includes Specialized Pro-resolving lipid Mediators (SPMs), a class of highly bioactive compounds known for their potent anti-inflammatory and pro-resolving effects [24,25]. These molecules, which include E-series and D-series Resolvins, protectins, and maresins, are biosynthesized from polyunsaturated fatty acids such as eicosapentaenoic acid (EPA) and docosahexaenoic acid (DHA) [26]. Resolvins of the D-series (RvDs), which are derived from DHA through enzymatic processes involving 15-lipoxygenase (15-LOX) and 5-lipoxygenase (5-LOX), have shown strong potential as natural antioxidants (Figure 3) [27]. Resolvin D1 (RvD1) and Resolvin D2 (RvD2), two well-studied members of this class, not only regulate ROS levels but also modulate immune responses by inhibiting the production of pro-inflammatory cytokines and promoting the M2 phenotype in macrophages, which is associated with tissue repair and inflammation resolution [28,29,30]. These features make RvDs attractive candidates for replacing synthetic antioxidants. However, their clinical application is currently limited by chemical instability and rapid metabolic inactivation. To address these limitations, researchers are developing stable analogs with enhanced pharmacological properties, aiming to improve the therapeutic potential of RvDs as safe and effective natural antioxidant agents [31].

Currently, there is a limited body of literature on the antioxidant and enzyme inhibition properties of Resolvin derivatives. In this study, we report for the first time the antioxidant activities of RvD1, RvD2, and their methyl ester derivatives using a range of in vitro antioxidant assays. To evaluate the total antioxidant capacity and activity of these molecules, several assays were employed: oxygen radical absorbance capacity (ORAC), hydroxyl radical scavenging (HRS), 2,2-diphenyl-1-picrylhydrazyl (DPPH), ferric reducing antioxidant power (FRAP), superoxide dismutase (SOD), and 2,2-azobis(2-amidinopropane) dihydrochloride (ABTS) assays. Additionally, hyaluronidase (HAase) inhibition assay was performed to investigate their potential role in OA treatment. The effect of RvD1, RvD2, and their methyl ester derivatives on HA protection was also investigated by incubating HMW HA, with ROS and checked by agarose gel electrophoresis and gel permeation chromatography.

## 2. Materials and Methods

### 2.1. Materials

Chemical reagents including fluorescein sodium salt (NaFluo, BioReagent), 2,2′-azobis(2-methylpropionamidine) dihydrochloride (AAPH, granules, 97%), 2,2-diphenyl-1-picrylhydrazyl (DPPH, powder), 2,2′-azino-bis(3-ethylbenzothiazoline-6-sulfonic acid) diammonium salt (ABTS, powder, 98%), potassium tetraborate tetrahydrate, potassium persulfate (powder, 99%), and the Superoxide Dismutase (SOD) Assay Kit were all purchased from MilliporeSigma Co (St. Louis, MO, USA). The Ferric Reducing Antioxidant Power (FRAP) Assay Kit (Colorimetric) was obtained from Thermo Fisher Scientific (Frederick, MD, USA). Antioxidant standards including (±)-6-hydroxy-2,5,7,8-tetramethylchromane-2-carboxylic acid (Trolox, 97%), (−)-Epigallocatechin gallate (EGCG), Quercetin, Ascorbic acid, and β-Carotene were also obtained from MilliporeSigma Co (St. Louis, MO, USA). High molecular weight sodium hyaluronate powder (>2000 kDa was obtained from Albomed GmbH (Schwarzenbruck, Germany). Hyaluronidase (50,000 U; bovine testes origin; activity ≥300 U mg^−1^) was obtained from STEMCELL Technologies (Vancouver, BC, Canada). RvD1 (7S,8R,17S-trihydroxy 4Z,9E,11E,13Z,15E,19Z-docosahexaenoic acid), RvD2 (7S,16R,17S-trihydroxy-4Z,8E,10Z,12E,14E,19Z-docosahexaenoic acid), and their corresponding methyl ester derivatives were purchased from Cayman Chemical Co. (Ann Arbor, MI, USA) and stored at −80 °C.

Preparation and Handling of Resolvin Derivatives: For all antioxidant assays, aliquots of Resolvin ethanolic stock solutions were diluted directly into assay-specific buffers to achieve the target final concentrations. To ensure chemical stability and minimize oxidative degradation, all procedures were performed under reduced light. Solutions were prepared fresh, protected from light during incubation, and utilized immediately following dilution to maintain the integrity of the lipid mediators. For enzyme inhibition assays, ethanol was removed under a gentle stream of nitrogen prior to reconstitution in acetate buffer.

### 2.2. Antioxidant Activity Assays

#### 2.2.1. Oxygen Radical Absorbance Capacity (ORAC) Assay

The ORAC assay was performed as reported literature. In short, a fresh stock solution of fluorescein (58 nM, in PBS) was prepared in a tube and stored at 4 °C. In a 96-well plate, 20 µL of different concentrations of Resolvins and controls were mixed with 120 µL of the fluorescein probe and incubated the mixture for 30 min at 37 °C. To establish the background signal, the initial fluorescence intensity (excitation. 485 nm, emission. 520 nm) was measured by fluorometer. Then, 60 µL of AAPH (40 mM) was added manually to all wells except for the negative control (fluorescein probe without AAPH). The plate was scanned in a microplate reader (Polar Star Optima, BMG Labtech, Ortenberg, Germany) for 250 cycles every 60 s. The final concentrations of all samples in the wells ranged from 0.625 to 20 µM. The Net AUC for Resolvins and controls were calculated and then plotted the values against the sample concentrations to create ORAC calibration curves. Trolox Equivalent (TE) was used to compare the antioxidant activity of all samples. The experiment was performed in triplicate.

#### 2.2.2. 2,2-Diphenyl-1-picrylhydrazyl (DPPH) Assay

DPPH method was performed with the method developed by Blois [32,33]. To start with, a fresh stock solution of DPPH reagent (600 µM) was prepared in methanol and stored at 4 °C. In a 96-well plate, 50 µL of different concentrations of Resolvins and controls were mixed with 50 µL of DPPH solution followed by incubating in a dark condition at room temperature for 30 min (final concentrations of all samples in the wells ranged from 0.625 to 20 µM). The absorbance was measured by the UV-Vis spectrophotometer at 517 nm and the scavenging percentage of all samples were plotted against the antioxidant concentrations to determine the IC_50_ (the concentration of the sample required to scavenge 50% of DPPH free radicals). The experiment was performed in triplicate.

#### 2.2.3. Ferric Reducing Antioxidant Power (FRAP) Assay

This experiment was conducted to measure antioxidant capacity using the FRAP assay and it was performed according to the FRAP kit protocol (MAK509-1KT Sigma Aldrich, St. Louis, MO, USA) [34]. In a 96-well plate, 25 µL of various concentrations of Resolvins and controls (ranged from 0.625 to 20 µM) were mixed with 75 µL FRAP color solution and incubated at room temperature for 30 min. After incubation, the absorbance of the mixture was measured at 593 nm using a UV spectrophotometer. According to the FRAP kit protocol, to quantify the antioxidant capacity, a calibration curve was plotted using a serial dilution of ferrous chloride (FeCl_2_) standard.

#### 2.2.4. 2,2-Azobis(2-amidinopropane) Dihydrochloride (ABTS) Assay

ABTS method was based on the method of van den Berg et al. [35] and slightly modified by Kim et al. [36] with some modification. Briefly, 7 mM of ABTS solution was prepared in PBS. ABTS radical cation was generated by mixing ABTS solution with the final concentration of 2.45 mM of potassium persulfate. The mixture was incubated for 12–24 h at dark at room temperature, then the absorbance of reaction mixture was adjusted to 0.70 ± 0.02 at 732 nm. In a half-area 96-well plate, 20 µL of different concentrations of Resolvins and controls were mixed with 80 µL of radical cation ABTS solution for 5 min, then absorbance of reaction mixture was measured at 734 nm for 20 min. All measurements were performed in triplicate. The scavenging activity and Trolox equivalent antioxidant capacity (TE) of samples were determined using the following equations:

ABTS^•+^ Scavenging% = [(Abs_Control_ − Abs_Sample_)/(Abs_Control_)] × 100

TE = Slope Sample/Slope Trolox

#### 2.2.5. Superoxide Dismutase (SOD) Assay

This experiment was conducted to measure antioxidant capacity to reduce superoxide radical and it was performed according to the SOD assay kit protocol (19160-1KT-F Sigma Aldrich). In a 96-well microplate, 20 µL of the sample solution was added to the designated Sample and Blank 2 wells, and 20 µL of ultrapure H_2_O to the Blank 1 and Blank 3 wells. Subsequently, 200 µL of WST Working Solution was added into all wells and mixed thoroughly. To Blank 2 and Blank 3 wells, 20 µL of Dilution Buffer was added. Finally, 20 µL of Enzyme Working Solution was added into the Sample and Blank 1 wells and mixed again. The plate was incubated at 37 °C for 20 min and the absorbance was measured at 450 nm using a microplate reader. The SOD activity was calculated as the percentage inhibition rate using the formula below:

SOD Activity (inhibition Rate%) = [(Abs_Blank1_ − Abs_Blank3_) − (Abs_Sample_ − Abs_Blank2_)]/(Abs_Blank1_ − Abs_Blank3_) × 100

#### 2.2.6. Hydroxyl Radical Scavenging (HRS) Assay

The HRS activity of Resolvins and controls was assessed in 96-well black plate under acellular conditions in the presence of H_2_O_2_ and CuCl_2_, as described in our previous study [31]. Briefly, ROS were generated in black 96-well plate by the addition of CuCl_2_ (10 mM) to the H_2_O_2_ (100 mM) prepared in PBS (75 mM, pH 7.4) in the presence or absence of 10 mM of the test compounds. After incubation for 120 min, the DCFH-DA probe was added to the mixture at a final concentration of 10 µM. Fluorescence was measured after an additional 30 min of incubation using a microplate reader equipped for fluorescence polarization (Polar Star Optima, BMG Labtech) set at an excitation wavelength of 485 nm and an emission wavelength of 530 nm. Results were expressed as relative fluorescence units (RFU).

#### 2.2.7. Hyaluronic Acid Degradation Study by Agarose Electrophoresis

The HA degradation assay was performed as described in our previous report, with minor modifications [31]. Briefly, a solution of HA (1 mg/mL; molecular weight > 2000 kDa) was treated with 10 µM CuCl_2_ and 100 µM H_2_O_2_ in the presence or absence of Resolvins, as well as controls, at a final concentration of 10 µM. The reaction mixture was incubated at 37 °C for 16 h. Subsequently, 10 µg of HA was loaded onto a 2% agarose gel prepared in Tris–borate–EDTA (TBE) buffer using a loading buffer containing 0.25% bromophenol blue dissolved in formamide. Electrophoresis was performed at 60 V for 4 h, and HA bands were visualized by staining with Stain-All dissolved in 30% ethanol.

#### 2.2.8. Hyaluronic Acid Analysis by Gel Permeation Chromatography

Gel permeation chromatography/size exclusion chromatography (GPC/SEC) analyses were conducted using two AquaGel columns (SB-806-M + SB-802; PolyAnalytik, London, ON, Canada) connected in series. The system consisted of a Viscotek TDA305 coupled with a GPCmax unit, equipped with an oven housing three detectors: a Refractive Index (RI) detector, Right-Angle and Low-Angle Light Scattering (RALS/LALS) detectors, and a four-capillary differential viscometer. Measurements were performed using 8.5 g/L NaCl in Milli-Q water as the mobile phase at 35 °C with a flow rate of 0.7 mL/min. Poly(ethylene glycol) (PEG, 20 kDa; dn/dc = 0.132 mL/g) and dextran (72 kDa; dn/dc = 0.147 mL/g) standards at 1.5–3.0 mg/mL were used to establish the calibration curve. Samples were prepared as Section 2.2.5. and then diluted in the mobile phase to approximately 0.25 mg/mL and filtered through 0.22 µm nylon syringe filters before injection. The original sample concentration was about 1 mg/mL, and the injection volume was 100 µL.

#### 2.2.9. Anti-Hyaluronidase Assay

Hyaluronidase inhibitory activity was assessed using a modified Morgan–Elson method [37]. Stock solutions of RvD1, RvD2, and their methyl-ester derivatives in ethanol were evaporated to dryness under a gentle stream of nitrogen at room temperature to remove solvent. The dried residues were reconstituted in 0.1 M acetate buffer (pH 3.6) and serially diluted to the final test concentrations (1.25–10 µM) immediately prior to the assay; samples were protected from light and handled quickly to minimize oxidation. The assay was performed using a 1 mg/mL HAase stock (≥300 Units/mL) prepared in 0.1 M acetate buffer (pH 3.6). For each test, 25 µL of sample was combined with 10 µL of the HAase stock and incubated at 37 °C for 20 min. Next, 20 µL of 12.5 mM CaCl_2_ was added to activate the enzyme and incubation continued at 37 °C for another 20 min. Subsequently, 25 µL of 3.5 mg/mL HA (in the same acetate buffer) was introduced and the mixture was incubated at 37 °C for 40 min. Reactions were terminated by adding 20 µL of 0.4 N NaOH followed by 20 µL of 0.4 N potassium tetraborate, and the tubes were heated at 100 °C for 3 min. After cooling to room temperature, 160 µL of 4-(dimethylamino) benzaldehyde reagent (final concentration 0.335 M) was added. Samples were incubated at 37 °C for 10 min and absorbance was measured at 585 nm. EGCG was used as a positive control. The HAase inhibition activity of samples was determined using the following equations:

HAase inhibition% = [(Abs_control_ − Abs_sample_)/(Abs_control_)] × 100

## 3. Results and Discussions

**Figure 4:**
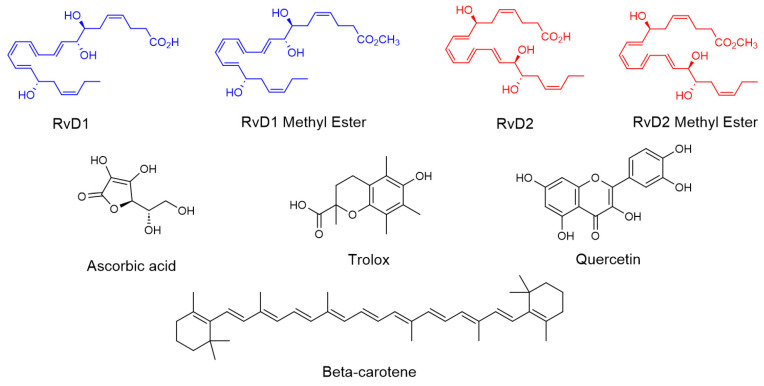
Chemical structure of RvD1, RvD2, and their methyl ester and all controls including Trolox, Ascorbic acid, β-Carotene, and Quercetin.

In this study, we investigated the antioxidant and enzyme inhibitory properties of four Resolvin derivatives (RvD1, RvD2, and their methyl ester) in comparison with four well-known antioxidant compounds (Trolox, β-Carotene, Ascorbic acid, and Quercetin) (Figure 4). Various in vitro chemical assays were employed to evaluate and compare their free radical scavenging and ROS reducing capacities. Additionally, we examined the potential of these Resolvins to inhibit key enzymes such as HAase, which is involved in the degradation of HA. The objective was to assess whether these bioactive lipid mediators could serve as promising candidates for protecting HA from degradation caused by both ROS and enzymatic activity in the synovial fluid—an important consideration for OA therapy.

### 3.1. Antioxidants and Radical Scavenging Activities of Resolvin Derivatives

#### 3.1.1. ORAC Assay

**Figure 5:**
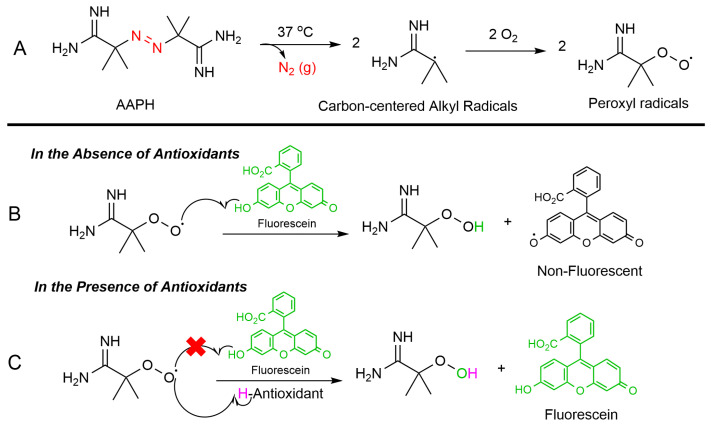
ORAC assay mechanism: (**A**) Thermal breakdown of AAPH in the presence of oxygen forms alkoxy radicals (ROS). (**B**) Without antioxidants, the peroxyl radicals degrade fluorescein, reducing its fluorescence. (**C**) Antioxidants neutralize ROS and preserve fluorescein’s fluorescence.

The ORAC assay is a widely used method to measure the antioxidant capacity of samples in various fields such as food analysis (to assess the antioxidant properties of fruits, vegetables, and other food products), pharmaceutical research (to evaluate the antioxidant activity of natural compounds and pharmaceutical drugs), and cosmetic industry (to determine the antioxidant benefits of skincare products) [38,39]. The ORAC assay relies on free radical damage to a fluorescent probe, most commonly fluorescein (FL), caused by an oxidizing reagent resulting in a loss of fluorescent intensity over time. This assay provides a quantitative measure of an antioxidant’s ability to inhibit free radical (peroxyl radical) which is produced by thermal decomposition of 2,2′-azobis(2-amidinopropan) dihydrochloride (AAPH) in the presence of oxygen. This process leads to the formation of nitrogen gas (N_2_) and two carbon-centered alkyl radicals (R^•^). Subsequently, these radicals may either rapidly recombine in a termination reaction or react with oxygen, forming peroxyl radicals (ROO^•^). The produced peroxyl radical can oxidize the fluorescein (FL), the fluorescence of the solution decreases over time. However, in the presence of an antioxidant, peroxyl radical can be scavenged, and the oxidation of fluorescein can be delayed until the antioxidant is depleted. Indeed, antioxidants protect fluorescein from ROS attacking via a hydrogen atom transfer (HAT) process (Figure 5).

**Figure 6:**
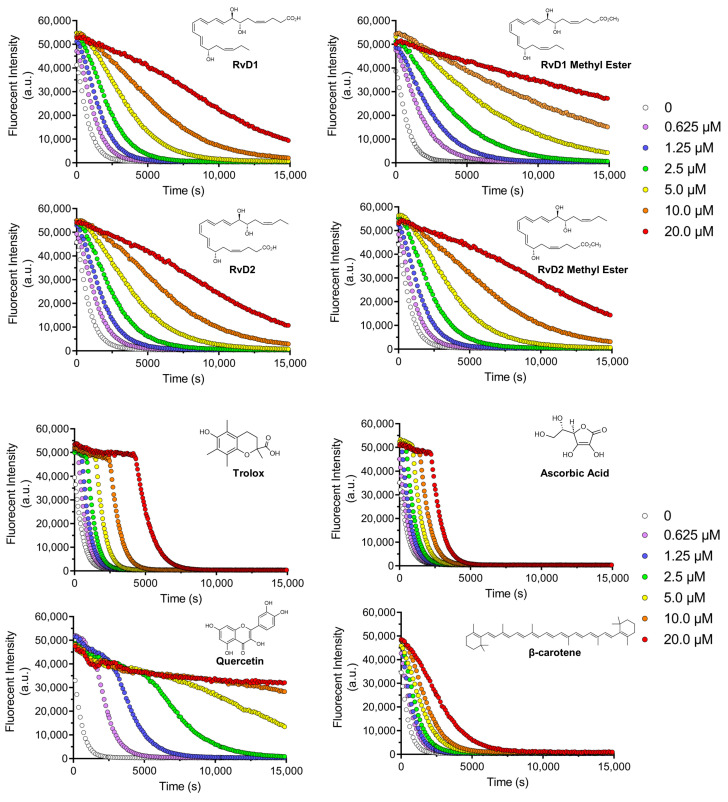
Decay curves for ORAC assay: Decay curves for different Resolvin samples (RvD1, RvD2, and their methyl ester derivatives) and four controls including Trolox, Ascorbic acid, Quercetin, and β-Carotene at different concentrations ranging from 0.625 to 20 µM.

Figure 6 illustrates the fluorescence decay of a probe in the absence and presence of increasing concentrations of RvD1, RvD2, and their corresponding methyl ester derivatives. All compounds displayed a dose-dependent antioxidant effect, as reflected by a slower decrease in fluorescence intensity over time. This suggests efficient scavenging of reactive species by these Resolvins and their derivatives. Notably, the RvD1 methyl ester exhibited the most antioxidant activity across all tested concentrations, maintaining higher fluorescence intensity throughout the experimental period. This effect was particularly at lower concentrations, indicating that methylation of the carboxylic acid group may enhance the compound’s lipophilicity, thereby improving its radical-scavenging performance. Similarly, RvD2 methyl ester showed superior antioxidant activity relative to RvD2, although the difference between these two was less pronounced than in the case of RvD1. As seen, the shape of the fluorescence ORAC decays does not present a clear distinction between the lag time and FL decay periods, which may indicate more complex scavenging kinetics.

Four controls of antioxidants with different properties and mechanisms of actions, including Trolox, Quercetin, Ascorbic acid, and β-Carotene were chosen to compare the results with Resolvins (Figure 6). Trolox and Ascorbic acid, two widely used hydrophilic antioxidants, demonstrated rapid and potent fluorescence preservation. The occurrence of a plateau along with the initial fluorescence measurement confirmed that these compounds were faster reactants towards AAPH-derived radicals compared to FL. Their characteristic profiles reflect strong free-radical scavenging abilities but with relatively short-lasting effects, as the fluorescence decays still tended to drop off sharply after the initial stabilization phase. In contrast, β-Carotene, a lipophilic antioxidant, showed a more gradual and concentration-dependent protective effect, though at higher doses (10.0–20.0 µM), its efficacy approached that of Trolox and Ascorbic acid. This is consistent with β-Carotene’s known mechanism of singlet oxygen quenching and its limited solubility, which may slow its interaction with the fluorescent probe. Quercetin displayed a distinctive antioxidant profile while its activity was clearly dose-dependent, high concentrations (10.0 and 20.0 µM) paradoxically led to a reduced protective effect. This might be attributed to Quercetin’s redox cycling behavior, where it can act as a pro-oxidant under certain conditions [40].

By comparing the controls and all Resolvins, the methyl esters of RvD1 and RvD2 displayed a particularly promising profile. RvD1 methyl ester showed protective activity comparable to Trolox and Ascorbic acid at concentrations as low as 1.25 µM, but with a more sustained fluorescence signal over time. This suggests that the methyl ester form may have longer-lasting radical scavenging capacity. Similarly, RvD2 methyl ester showed moderately enhanced activity compared to its acid form, though its overall antioxidant potency was lower than that of RvD1 methyl ester.

In addition to the fluorescence decay kinetics, the antioxidant activity of all compounds was further quantified using Trolox equivalents (TE), providing a standardized measure for direct comparison. As summarized in Table 1, among the tested antioxidants, Quercetin exhibited the highest TE value of 5.48 ± 0.13, confirming its strong radical-scavenging activity. Trolox, as the reference standard, was assigned a TE value of 1, while Ascorbic acid and β-Carotene showed lower TE values of 0.65 ± 0.01 and 0.56 ± 0.05, respectively, reflecting their moderate but consistent antioxidant capacities. Interestingly, RvD1 methyl ester demonstrated a TE value of 3.49 ± 0.3, ranking second only to Quercetin among all compounds tested, and significantly surpassing Trolox. This supports its superior performance observed in the fluorescence decay assay. Similarly, RvD2 methyl ester (1.45 ± 0.05) and RvD2 (1.38 ± 0.02) outperformed Trolox, further emphasizing their promising antioxidant potential. In comparison, RvD1 (1.22 ± 0.1) also exceeded Trolox, although its activity was lower than its methyl ester derivative and the RvD2 compounds.

The ranking of antioxidant capacity based on TE values can therefore be summarized as:

Quercetin > RvD1 methyl ester > RvD2 methyl ester > RvD2 > RvD1 > Trolox > Ascorbic acid > β-Carotene.

#### 3.1.2. DPPH Assay

The DPPH assay was included to allow comparison with commonly used reference antioxidants, despite its known limitations when applied to lipid mediators like Resolvins. This assay relies on a direct electron- or hydrogen-donating mechanism and is generally more suitable for small, hydrophilic antioxidants, while lipid-derived mediators often show poor reactivity due to limited solubility and weak interaction with the DPPH radical. In our DPPH assay, neither Resolvin D1 nor D2, including their methyl ester derivatives, showed very low activity against the DPPH radical (not higher than 2.8%) for the highest final concentration of 20 µM. This low reactivity may be attributed to their poor interaction with the DPPH radical under the tested conditions. Extending the reaction time to 120 min did not result in a significant decrease in DPPH absorbance, further confirming the absence of interaction. The results are summarized in Table 2. Trolox—used as a positive control—exhibited radical scavenging percentage of 20.496% at 20 µM. The other controls were also tested and, both Quercetin and Ascorbic acid demonstrated 42.75, and 13.24% scavenging activity (at the highest tested concentration of 20 µM). However, surprisingly, β-Carotene as a control showed lack of activity in scavenging DPPH radical.

This lack of activity for β-Carotene is consistent with previous findings. For instance, Müller et al. [41], reported the antioxidant activities of several carotenoids under standardized conditions and surprisingly, neither the carotenes nor the xanthophylls showed any DPPH radical scavenging activity. Taken together, these findings indicate that the DPPH assay is not well suited for evaluating the antioxidant potential of lipid mediators such as Resolvin D1, Resolvin D2, and their derivatives. Their minimal activity is most likely attributable to limited electron-donating capacity and weak interaction with the DPPH radical, rather than the absence of antioxidant properties. This underscores the necessity of employing complementary antioxidant assays that are more compatible with lipid-derived bioactive molecules.

#### 3.1.3. FRAP Assay

The FRAP assay was employed to assess the electron-donating capacity of the tested compounds via a single-electron transfer (SET) mechanism. While FRAP is widely used for phenolic and water-soluble antioxidants, its applicability to lipid mediators is limited due to structural and mechanistic constraints, including the requirement for efficient electron transfer under acidic conditions. The FRAP assay evaluates antioxidant capacity by measuring the ability of compounds to reduce the Fe^3+^-TPTZ (ferric-tripyridyltriazine) complex to its Fe^2+^ form, which yields an intense blue color measurable at 593 nm [42]. FRAP operates via a SET mechanism, offering complementary insight into the electron-donating properties of antioxidant compounds [43,44]. All compounds were evaluated for their FRAP activity over a concentration range of 0.625–20 µM. The results reported in Table 3 reflect Fe^2+^ concentrations (µM) generated at the highest tested concentration (20 µM) of each antioxidant, representing their maximal ferric-reducing capacity under the assay conditions. Trolox and Ascorbic acid, used as positive controls, showed strong reducing power with Fe^2+^ equivalent concentration of 229 ± 5.14 and 262 ± 5.46 µM, respectively, confirming their well-established activities. Quercetin also demonstrated significant reducing abilities, yielding 618 ± 9.18 µM of Fe^2+^ equivalents. In contrast, β-Carotene displayed only negligible antioxidant effects in this assay. Similarly, all Resolvin compounds—RvD1 (6.1 ± 0.32 µM), RvD1 methyl ester (7.5 ± 0.96 µM), RvD2 (5.9 ± 0.64 µM), and RvD2 methyl ester (9.7 ± 0.32 µM)—exhibited minimal ferric-reducing activity at all tested concentrations. While Trolox, Quercetin, and Ascorbic acid reacted almost instantaneously, the Resolvin compounds and β-Carotene showed only a minimal increase in absorbance over 30 min. Extending the incubation time to three hours did not result in any significant increase in Fe^2+^ production, suggesting a genuine lack of reducing capability rather than slow kinetics.

Previous studies by Pulido et al. [45] reported that polyphenols, Trolox, Ascorbic acid, Tannic acid, and several other antioxidants exhibited strong FRAP activity, whereas resveratrol showed minimal reducing power, and carotenoids such as β-Carotene and zeaxanthin lacked ferric-reducing ability. In contrast, Müller et al. [41] demonstrated that several carotenoids do possess FRAP activity, with lycopene exhibiting higher ferric-reducing potential than even α-tocopherol. This activity was shown to be strongly dependent on the extent of the conjugated double bond (CDB) system and the presence of hydroxyl groups, with structural features such as the β-ionone ring and epoxidation further modulating activity via steric or electronic effects [41]. The absence of measurable FRAP activity in Resolvins may be attributed to their structural characteristics, which lack the extensive conjugated π-electron systems typically required for effective electron transfer. Resolvins also contain multiple C=C bonds, but they are not conjugated across the molecule. This interrupted conjugation means “Resolvin radicals intermediate” cannot delocalize charge over a large π-system. Moreover, the redox potential of a compound influences its ability to reduce ferric ions. Since the redox potentials of RvD1, RvD2, and their derivatives are not yet well characterized, further electrochemical studies are required to confirm whether their inactivity is due to a fundamentally low electron-donating capability or an incompatibility with the FRAP assay conditions (e.g., acidic pH, presence of organic solvents).

Overall, these results demonstrate that FRAP, like DPPH, is poorly suited for assessing the antioxidant properties of lipid mediators such as Resolvins. Their minimal activity in this assay should therefore be interpreted as a limitation of the assay methodology rather than definitive evidence of low antioxidant potential.

#### 3.1.4. ABTS Assay

**Figure 7:**
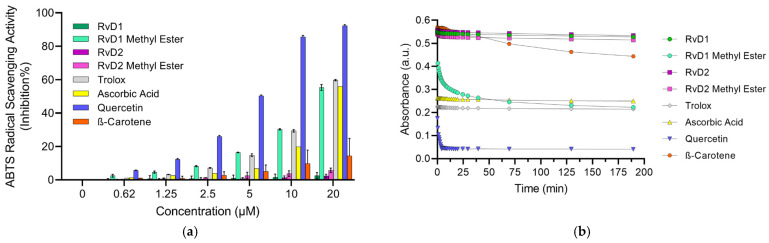
ABTS assay for different Resolvin samples and controls: (**a**) Concentration-response graph for ABTS^•+^ scavenging activity of all Resolvins and controls at 734 nm. (**b**) Absorbance of ABTS^•+^ solution mixed with antioxidants (20 µM) at different time. Data are presented as mean ± SD (n = 3).

The ABTS antioxidant assay is a widely used method for evaluating the antioxidant capacity of various substances, including plant extracts, food, clinical fluids [46]. This technique involves the generation of the stable ABTS^•+^ radical cation, a blue-green chromophore with absorption maxima at 734 nm. The radical cation is typically produced by reacting ABTS with a strong oxidizing agent, such as potassium persulfate (K_2_S_2_O_8_) [43,46]. Once formed, ABTS^•+^ can be readily reduced by antioxidants, leading to a decrease in absorbance at 734. The extent of decolorization, measured as a percentage inhibition of the ABTS^•+^ radical cation, is directly proportional to the antioxidant activity of the sample [47]. Likewise, DPPH assay, the time of various antioxidants quenching ABTS^•+^ is different because ABTS^•+^ could be quenched by both electron (fast) and hydrogen atom transfer (slow). Our results revealed that ABTS^•+^ could be quenched very rapidly by Trolox with an inhibition of 59.7% like Ascorbic acid with an inhibition percentage of 55.94% at the tested concentration of 20 µM. Quercetin as another control showed a very high inhibition of 92.41% (Figure 7a, Table 4). On the other hand, β-Carotene showed a moderate scavenging activity (22.27%, at 20 µM). Interestingly, among the tested Resolvins—RvD1, RvD2, and their methyl ester derivatives—only RvD1 methyl ester exhibited notable antioxidant activity, showing a scavenging capacity comparable to that of Trolox (Figure 7a, Table 4). This result is particularly surprising, given that RvD1 methyl ester, like other Resolvins, did not show any activity in the DPPH or FRAP assays. This result suggests that RvD1 methyl ester may possess a unique structural feature or mechanism that enables efficient electron or hydrogen donation in the ABTS^•+^ assay, despite its lack of activity in two previous antioxidant assays. Further investigation is warranted to elucidate the underlying molecular interactions and to better understand their potential as a selective antioxidant in biological systems.

All the above-mentioned observations are also calculated by the Trolox Equivalent (TE) values (Table 5). Quercetin exhibited the highest TE value (3.65), indicating a much stronger antioxidant capacity than Trolox (TE = 1.00). Ascorbic acid showed equivalent antioxidant capacity to Trolox (TE = 1.01), while β-Carotene exhibited a moderate TE value of 0.47. Among the Resolvins, RvD1 methyl ester showed the highest TE value (0.92), suggesting moderate antioxidant capacity, whereas RvD1, RvD2, and RvD2 methyl ester displayed very low TE values (0.02, 0.036, and 0.095, respectively), aligning with their weak and slow ABTS^•+^ scavenging behavior observed in the kinetic study.

These observations underscore the need to evaluate ABTS^•+^ quenching in a time-dependent manner rather than relying solely on single-point measurements. Dong et al. [48] suggested that ABTS^•+^ assay could be measured within 10 min to obtain a rough result; however, full and accurate evaluation of antioxidant reactivity rather than capacity requires recording ABTS^•+^ loss continuously during the whole reaction period. Hence, the ABTS^•+^ scavenging activity of all samples were determined under different reaction times. As illustrated in Figure 7b, Trolox, Quercetin, and Ascorbic acid show a steep decline in absorbance within the first few seconds, indicating fast and efficient ABTS^•+^ quenching. In contrast, β-Carotene displays a shallow slope, reflecting its slow reaction kinetics. Among the Resolvins, RvD1 methyl ester shows a relatively fast initial quenching followed by a slower phase, suggesting a biphasic reaction. RvD2 and its methyl ester, as well as RvD1, demonstrate lower scavenging activity, with minor changes in absorbance even after extended reaction times, highlighting their weak radical quenching ability.

#### 3.1.5. SOD Assay

The superoxide dismutase (SOD) assay is designed to quantify the ability of SOD or SOD-like antioxidants to neutralize the superoxide radical (O_2_^−•^), a ROS that contributes to oxidative stress and cellular damage. This indirect assay utilizes the water-soluble tetrazolium salt, WST-1 [2-(4-Iodophenyl)-3-(4-nitrophenyl)-5-(2,4-disulfophenyl)-2H-tetrazolium, monosodium salt], which generates water-soluble formazan dye upon reduction by superoxide anions [49]. In this system, xanthine oxidase catalyzes the oxidation of xanthine to uric acid, producing superoxide as a byproduct. The rate of this reaction is directly proportional to the antioxidant activity of samples since they can inhibit yellow formazan formation by reducing the superoxide anion radical. By measuring the decrease in color development at 440 nm, the SOD activity of samples can be quantified as an inhibition rate. The mechanism of reduction of superoxide anion radicals by antioxidants typically involves a single electron transfer (SET) reaction, where the antioxidant donates an electron to neutralize the radical.

**Figure 8:**
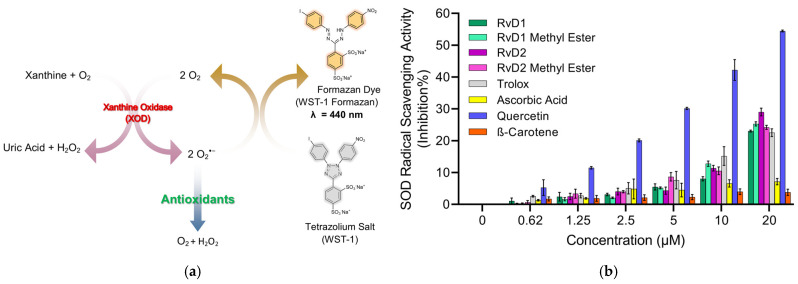
Principle and evaluation of SOD assay kit. (**a**) Schematic representation of the SOD assay principle. Superoxide anions (O_2_^−•^) are generated by the enzymatic reaction of xanthine and oxygen in the presence of xanthine oxidase (XOD). These radicals reduce the tetrazolium salt WST-1 to a water-soluble formazan dye (λ = 440 nm). Antioxidants inhibit this reduction by scavenging superoxide radicals, leading to reduced absorbance. (**b**) SOD radical scavenging activity (% inhibition) of various compounds, including RvD1, RvD1 methyl ester, RvD2, RvD2 methyl ester, Trolox, Ascorbic acid, Quercetin, and β-Carotene at different concentrations (0.62–20 µM). Data are presented as mean ± SD (n = 3).

The results (Figure 8) indicate that Quercetin exhibited the strongest superoxide scavenging activity, showing a clear dose-dependent response across all tested concentrations. This finding aligns with previous reports highlighting Quercetin’s highly efficient radical-scavenging properties, which are attributed to its polyhydroxylated flavonoid structure providing multiple active sites for electron donation. Trolox also demonstrated strong superoxide scavenging activity, further validating the assay and confirming its role as a reliable positive control. By contrast, Ascorbic acid displayed only modest inhibition at lower concentrations, but at higher doses its scavenging activity became comparable to that of Trolox. Although Ascorbic acid is widely recognized for its antioxidant potential, its weaker performance in this assay may be explained by its preferential reactivity toward other radicals (such as hydroxyl or peroxyl) rather than superoxide. Furthermore, β-Carotene exhibited negligible SOD-like activity across all concentrations, which can be attributed to its poor solubility in aqueous systems and its lipophilic nature, limiting its ability to interact effectively with the hydrophilic superoxide radical in the assay medium.

Among the Resolvin samples, RvD1 methyl ester showed SOD-like activity, comparable to Trolox. In contrast, RvD1, RvD2, and RvD2 methyl ester did not display measurable SOD-like activity. This outcome is consistent with the ABTS assay results, where RvD1 methyl ester also demonstrated higher radical scavenging activity compared to the other Resolvin derivatives. These findings suggest that structural modifications, such as esterification, may enhance the radical scavenging properties of certain Resolvin derivatives, highlighting the unique antioxidant potential of RvD1 methyl ester.

#### 3.1.6. HRS Assay

**Figure 9:**
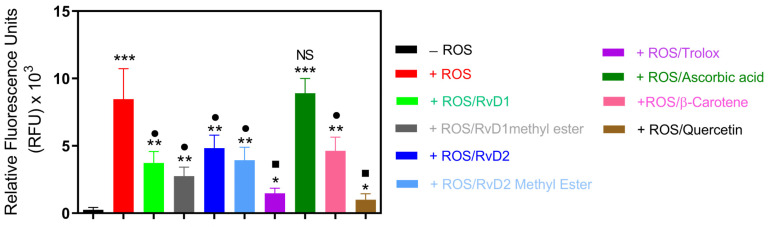
ROS induced by a CuCl_2_/H_2_O_2_ system were treated with RvD1, RvD2, their methyl esters, and known antioxidant standards (Trolox, Ascorbic acid, β-Carotene, and Quercetin). The intensity of ROS generation was quantified spectrophotometrically and expressed as a percentage relative to the ROS control group. All tested compounds reduced ROS formation except Ascorbic acid. Data are presented as mean ± SD (n = 3). * *p* < 0.05, ** *p* < 0.01, *** *p* < 0.001 (vs. CTL); • *p* < 0.05, ▪ *p* < 0.01, (vs. ROS), NS (not significant).

This experiment was designed to investigate the antioxidant properties of RvD1, RvD2, and their methyl ester using CuCl_2_ + H_2_O_2_-induced ROS generation in a cell-free system, as described in our previous study [31]. This model allowed direct comparison of their radical scavenging activities with established controls. As shown in Figure 9, exposure to CuCl_2_/H_2_O_2_ markedly increased ROS generation in the control group. The addition of RvD1, RvD1 methyl ester, RvD2, RvD2 methyl ester, Trolox, β-Carotene, and Quercetin resulted in significant suppression of ROS formation, reducing ROS levels by approximately 56%, 67%, 43%, 53%, 82%, 45%, and 88%, respectively. Among the tested compounds, Quercetin exhibited the highest antioxidant efficacy, followed closely by Trolox. In contrast, Ascorbic acid did not display a significant inhibitory effect under these conditions, suggesting limited scavenging efficiency in this specific oxidative model.

Based on the calculated radical scavenging percentage, the relative antioxidant capacities can be ranked as follows: Quercetin > Trolox > RvD1 methyl ester > RvD1 > RvD2 methyl ester > β-Carotene > RvD2 > Ascorbic acid. Interestingly, the methyl ester derivatives of both RvD1 and RvD2 demonstrated enhanced antioxidant capacity compared to their acid forms, confirming other radical scavenging tests.

#### 3.1.7. Evaluation of Hyaluronic Acid Oxidative Degradation

##### Agarose Gel Electrophoresis

In vivo, the limitation use of HA is attributed to its instability and susceptibility to degradation by the attack of free radicals and the enzymatic action of HAase. To address this, numerous chemical modification methods have been proposed to reduce the rate of HA degradation and thus prolong its lifespan in the joint after injection. To further elucidate the antioxidant properties of RvD1, RvD2, and their methyl ester derivatives, their ability to protect HA from degradation induced by ROS was investigated. For this purpose, high–molecular weight HA polymer was first incubated with each compound and subsequently exposed to a pro-oxidant system composed of CuCl_2_ and H_2_O_2_ for 16 h. This combination generates hydroxyl radicals capable of cleaving HA chains, thereby serving as a model to assess oxidative degradation. Following treatment, the extent of HA degradation was analyzed by agarose gel electrophoresis, and the bands were visualized using stain-all solution, a dye specific for glycosaminoglycans. This method enabled a qualitative comparison of HA integrity among the different treatment groups.

**Figure 10:**
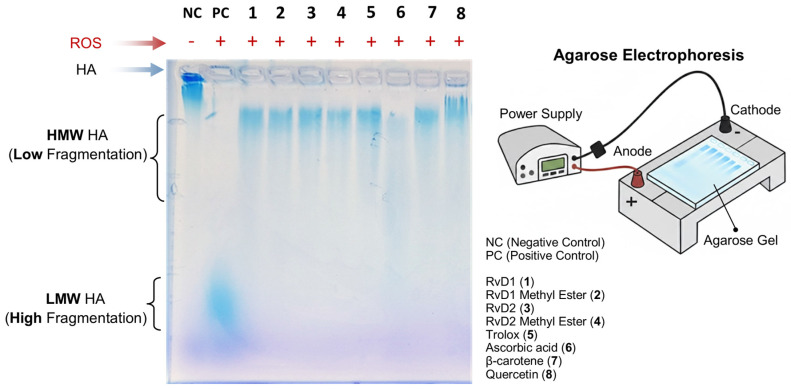
Evaluation of hyaluronic acid (HA) protection against reactive oxygen species (ROS) by RvD1, RvD2, and their methyl esters using agarose gel electrophoresis. HA samples were incubated with ROS in the presence or absence of Resolvins derivatives and antioxidant controls. The electrophoresis image shows that RvD1, RvD2, and their methyl esters effectively protected HA from degradation, maintaining a high molecular weight (HMW HA, low fragmentation). Similar protective effects were observed with Trolox, β-Carotene, and Quercetin, whereas Ascorbic acid exhibited moderate HA protection. In contrast, the HA + ROS sample (positive control) displayed extensive degradation, corresponding to low molecular weight HA (LMW HA, high fragmentation) migrating toward the bottom of the gel.

As shown in Figure 10, the addition of RvD1, RvD2, and their methyl ester forms attenuated CuCl_2_/H_2_O_2_-induced HA fragmentation, preserving the polymer’s structural integrity relative to untreated samples. The protective effect observed suggests that these compounds possess notable ROS-scavenging capability, preventing the oxidative breakdown of HA. Among the tested compounds, Quercetin, a well-known antioxidant, exhibited the highest degree of protection, serving as a positive control and confirming the reliability of the assay. These findings collectively support the hypothesis that RvD1 and RvD2, along with their derivatives, exert antioxidant actions capable of mitigating oxidative damage to extracellular matrix components such as HA.

We are keeping in mind that the in vitro results are often not representative of the results observed in vivo. This could be caused by the complexity of the natural biological environment that is missing in vitro. Thus, this represents one of the inevitable limitations of the present study. Our obtained data will be completed by investigating, in vitro, the enzymatic conversion of Resolvins and their derivatives by eicosanoids oxidoreductase and human liver microsomes as well as the biological activity of their metabolites. Furthermore, the biological effects of the formulation of HA need to be investigated in our future studies, including the expression of catabolic and inflammatory mediators in human cartilage explants and isolated chondrocytes from OA patients. To mimic the in vivo situation, additional in vitro experiments will be planned to explore the stability of HA mixed or not with resolvins and incubated with serum or synovial fluid from OA patients.

In physiological and pathological conditions, a number of preclinical and clinical studies will be performed in the future to determine, first, the biological effects of these formulations, their physicochemical properties in a biological environment as well as their biocompatibility, and second, to investigate the pharmacokinetic and pharmacodynamics (PK/PD) properties of the chemical compounds and their effects on the stability of HA. The findings will enable the use of these molecules to produce a next-generation viscosupplement for the treatment of OA, featuring excellent biocompatibility, extremely low toxicity, and efficacy in reducing oxidation and inflammation, while optimizing the functionalization potential of existing HA.

##### Gel Permeation Chromatography

**Figure 11:**
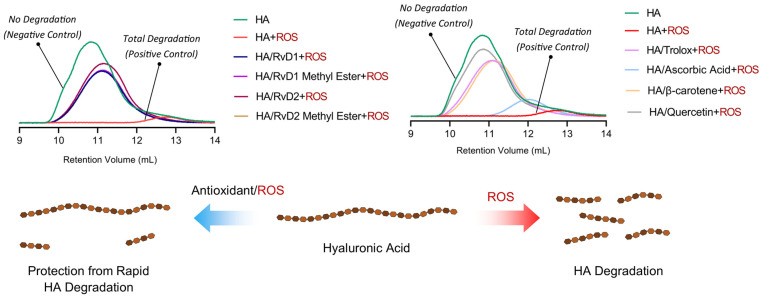
GPC/SEC chromatograms and schematic illustration showing the protective effects of Resolvin derivatives on HA degradation. The chromatograms compare molecular weight distributions of HA after oxidative stress induced by CuCl_2_/H_2_O_2_, with or without the presence of RvD1, RvD2, and their methyl ester derivatives, as well as reference antioxidants (Trolox, Ascorbic acid, β-Carotene, and Quercetin).

The degradation of HA under oxidative conditions was assessed to determine the protective effects of RvD1, RvD2, and their methyl ester derivatives (Figure 11). GPC/SEC chromatograms demonstrated a shift in retention volume and a drastic reduction in HA’s molecular weight when incubated with CuCl_2_/H_2_O_2_ alone, confirming extensive oxidative degradation. Quantitative analysis of molecular weight parameters corroborated the observations (Table 6). Specifically, the average molecular weight (M_w_) of HA dropped from 1,877,000 Da in control to only 19,221 Da upon exposure to CuCl_2_/H_2_O_2_, signifying near-complete depolymerization. In contrast, the co-incubation with RvD1, RvD2, and their methyl esters preserved within the 462,000–530,000 Da range, demonstrating substantial protection against oxidative cleavage. Among the reference antioxidants, Trolox and β-Carotene provided moderate protection, while Ascorbic acid exhibited relatively lower efficacy. Quercetin, however, showed particularly significant results in radical scavenging. Overall, these findings highlight the potent antioxidant activity of Resolvin derivatives as promising adjuncts for improving the stability and therapeutic durability of HA-based viscosupplements in OA.

Multiple antioxidant assays were conducted to provide a comprehensive evaluation of Resolvins’ radical-scavenging potential. DPPH and FRAP showed no measurable activity, likely due to limited solubility and weak electron transfer capacity of lipid mediators, rather than absence of antioxidant potential. Therefore, these assays are not emphasized. In contrast, activity was observed in ORAC, ABTS^+^, HRS, and SOD assays. RvD1 methyl ester exhibited the strongest effects in ORAC and ABTS^+^ scavenging, as well as in SOD and HRS assays. These results indicate selective antioxidant activity of Resolvins, particularly for peroxyl radicals, ABTS^+^, and superoxide, with minimal electron transfer activity in DPPH and FRAP assays.

Differences among RvD1, RvD2, and their methyl ester derivatives likely reflect both intrinsic chemical reactivity and physicochemical properties, including solubility, dispersion, and stability. The higher activity of methyl esters, especially RvD1 methyl ester, may result from increased lipophilicity, better dispersion, and reduced ionization or degradation. Weak responses in electron transfer assays appear assay-dependent, as similar behavior was observed for β-carotene, underscoring mechanism-specific limitations of DPPH and FRAP.

### 3.2. Hyaluronidase Enzyme Inhibition

HA, an abundant glycosaminoglycan in the extracellular matrix (ECM), is critical for tissue hydration, viscoelasticity, and joint lubrication. In healthy tissue, HA homeostasis is maintained by a tightly regulated balance between synthesis by HA synthases (HASs) and degradation by HAases. Excessive HAase activity, however, accelerates HA turnover and yields low-molecular-weight fragments that promote inflammation, cartilage breakdown, and pain—hallmarks of OA pathology [50]. In the context of intra-articular HA injection for OA therapy, rapid enzymatic degradation of the injected polymer severely limits residence time and clinical efficacy. Consequently, co-administration of HAase inhibitors—including small-molecule antioxidants and targeted enzyme inhibitors—has emerged as a promising strategy to stabilize HA in the joint space. By suppressing HAase activity, these compounds prolong the half-life of therapeutic HA, enhance its chondroprotective and anti-inflammatory actions, and may improve patient outcomes in OA. In this study, the inhibitory potency of RvD1, RvD2, and their methyl ester derivatives against HAase was also evaluated using Morgan-Elson-based colorimetric assay, and their potential to augment HA injection therapies in OA joints was discussed [51].

**Figure 12:**
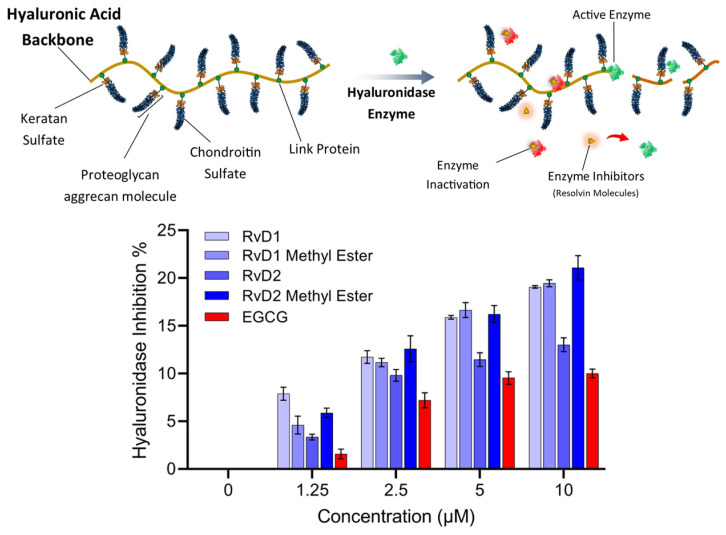
Schematic representation and experimental analysis of HAase enzyme inhibition by Resolvin compounds. (**Top**) Illustration of HA degradation by HAase, with Resolvin molecules (RvD1, RvD2, and their methyl esters) acting as enzyme inhibitors that prevent the breakdown of HA. (**Bottom**) Dose-dependent inhibition of HAase activity by RvD1, RvD2, their methyl ester derivatives, and EGCG, as a control. Data represent mean ± SD (n = 2).

As shown in Figure 12, all Resolvin derivatives produced a modest but measurable reduction in hyaluronidase activity in a concentration-dependent manner. At the highest tested concentration (10 µM), inhibition ranged from approximately 15% to 21%, with methyl ester derivatives showing slightly greater inhibition than their corresponding free acid forms. However, the overall level of inhibition remained below 25% across all compounds and concentrations tested. EGCG, which was selected as a reference compound due to its widespread use as an antioxidant and hyaluronidase inhibitor control in the literature, also exhibited limited inhibitory activity under the present assay conditions.

Overall, these results suggest that Resolvins exert partial inhibition of hyaluronidase activity rather than acting as potent enzyme inhibitors at the tested concentrations and pH. While the acidic pH used in this study maximizes enzymatic activity and assay sensitivity, it does not fully reflect the physiological conditions of human joint tissues, where hyaluronidase isoforms may operate at near-neutral pH and exhibit different kinetic behavior. Consequently, these results should be interpreted as a preliminary assessment of hyaluronidase modulation, and further studies involving multiple enzyme isoforms, physiological pH conditions will be required to define inhibitory potency and biological relevance more definitively.

## 4. Conclusions

This study demonstrates that RvD1, RvD2, and—most notably—their methyl ester derivatives exert dual protective actions on HA in vitro: (1) antioxidant-/radical-scavenging activities and (2) inhibition of HA-degrading enzyme activity. Across multiple antioxidant assays, RvD1 methyl ester consistently showed the strongest activity (e.g., an ORAC Trolox equivalent of 3.49 ± 0.30 and substantial ABTS- and SOD-like responses), whereas the RvD1 and RvD2 derivatives produced weaker, assay-dependent effects. Functionally, co-incubation of HA with ROS plus Resolvin derivatives preserved HA molecular weight in the 4.6 × 10^5^–5.2 × 10^5^ Da range compared with near-complete depolymerization (≈1.9 × 10^4^ Da) by ROS alone (positive control); similarly, Resolvin methyl esters produced greater hyaluronidase inhibition (~20–21%) than the polyphenolic control EGCG under the conditions tested. These results indicate that Resolvin derivatives can both reduce oxidative fragmentation of HA and slow enzymatic degradation, two important mechanisms that limit the residence time and performance of viscosupplementation. Although RvD1 and RvD2 are structural isomers, differences between them are likely reflect both intrinsic chemical reactivity and physicochemical properties, including solubility, dispersion, and stability. The higher activity of methyl esters, especially RvD1 methyl ester, may result from increased lipophilicity, better dispersion, and reduced ionization or degradation.

While RvD1 methyl ester demonstrated antioxidant activity in our study, we acknowledge that this effect is specific to certain radical scavenging mechanisms. Our data indicate superior performance in assays governed by HAT and those targeting superoxide-related species, rather than SET pathways. Consequently, the antioxidant capacity of RvD1 methyl ester should not be interpreted as a universal radical-scavenging property, but rather as a mechanism-specific intervention that may be particularly effective against oxidative stressors prevalent in the joint environment, such as superoxide anions. Furthermore, our in vitro results provide evidence that RvD derivatives can enhance HA stability against oxidative and enzymatic degradation, we acknowledge the inherent limitations of using only chemical, acellular, and enzyme-based assays. Specifically, our current findings do not fully address the complex biological milieu of the joint. Future research is required to evaluate factors critical for clinical efficacy, including the resident time, cellular clearance mechanisms, and in vivo pharmacokinetics of these derivatives. Our team is actively pursuing a dedicated study on these biological aspects, which will include assays, and these results will be reported in a separate publication. Taken together, our findings provide a positive preliminary answer to the central question of this study—whether Resolvin D1, D2, and their methyl esters can enhance the efficacy of hya-luronic acid injections in OA—while underscoring the need for further formulation and animal studies to confirm their therapeutic potential.

## Figures and Tables

**Table 1:** The Trolox Equivalent (TE) values antioxidant samples. Data represent mean ± SD (n = 3).

Antioxidant	RvD1	RvD1 Methyl Ester	RvD2	RvD2 Methyl Ester	β-Carotene	Quercetin	Ascorbic Acid	Trolox
**TE Value**	1.22 ± 0.1	3.49 ± 0.3	1.38 ± 0.02	1.45 ± 0.05	0.56 ± 0.05	5.48 ± 0.13	0.65 ± 0.01	1

**Table 2:** DPPH Radical Scavenging Activity of Resolvins and Controls at 20 µM concentration. Data represent mean ± SD (n = 3).

Antioxidant	RvD1	RvD1 Methyl Ester	RvD2	RvD2 Methyl Ester	β-Carotene	Quercetin	Ascorbic Acid	Trolox
**% Inhibition**	1.641 ± 0.6	1.998 ± 0.3	2.81 ± 0.8	1.408 ± 0.5	0.0 ± 0	42.75 ± 0.1	13.24 ± 0.3	20.496 ± 0.6

**Table 3:** Ferric-reducing antioxidant power (FRAP) of selected compounds at 20 µM concentration, expressed as Fe^2+^ equivalents (µM). Higher Fe^2+^ concentrations indicate stronger ferric reducing (antioxidant) activity. Resolvin compounds and β-Carotene showed minimal FRAP activity, while Trolox, Quercetin, and Ascorbic acid exhibited higher reducing capacities. Values represent mean ± standard deviation from three independent experiments. Data represent mean ± SD (n = 3).

Antioxidant	RvD1	RvD1 Methyl Ester	RvD2	RvD2 Methyl Ester	β-Carotene	Quercetin	Ascorbic Acid	Trolox
**Fe^2+^ Conc.** **(µM)**	6.1 ± 0.32	7.5 ± 0.96	5.9 ± 0.64	9.7 ± 0.32	1.5 ± 0.08	618 ± 9.18	262 ± 5.46	229 ± 5.14

**Table 4:** ABTS^•+^ scavenging activity of all samples and control at highest concentration (20 µM). Data are presented as mean ± SD (n = 3).

Antioxidant	RvD1	RvD1 Methyl Ester	RvD2	RvD2 Methyl Ester	β-Carotene	Quercetin	Ascorbic Acid	Trolox
**% Inhibition**	2.6% ± 1.8	55.3% ± 1.7	2.3% ± 1.1	5.7% ± 1.3	14.3% ± 10.5	92.4% ± 0.5	55.9% ± 2.3	59.7% ± 1.8

**Table 5:** The Trolox Equivalent (TE) values antioxidant samples.

Antioxidant	RvD1	RvD1 Methyl Ester	RvD2	RvD2 Methyl Ester	β-Carotene	Quercetin	Ascorbic Acid	Trolox
**TE Value**	0.02 ± 0.001	0.92 ± 0.02	0.036 ± 0.004	0.095 ± 0.001	0.47	3.65	1.01	1

**Table 6:** Molecular weight parameters of HA after oxidative degradation with H_2_O_2_ in the absence or presence of Resolvins and controls determined by GPC/SEC analysis. The table summarizes retention volume (V_p_), weight-average molecular weight (M_w_), number-average molecular weight (M_n_), polydispersity index (M_w_/M_n_), and refractive index increment (dn/dc). The results indicate that Resolvin derivatives, particularly their methyl ester forms, preserve HA molecular weight against complete oxidative degradation.

Samples	V_p _ (mL)	M_w _ (Da)	M_n _ (Da)	M_w_/M_n _ (-)	dn/dc (mL/mg)
HA	10.91	1,877,000	1,978,000	1.054	0.1292
HA + ROS	13.04	19,221	34,118	1.775	0.1126
HA/RvD1 + ROS	11.38	523,081	726,499	1.389	0.1086
HA/RvD1 methyl ester + ROS	11.38	490,951	681,433	1.388	0.1139
HA/RvD2 + ROS	11.46	466,835	634,524	1.359	0.1286
HA/RvD2 methyl ester + ROS	11.39	497,413	695,645	1.399	0.1123
HA/Trolox + ROS	11.35	588,214	800,961	1.362	0.1138
HA/Ascorbic acid + ROS	12.77	72,566	123,754	1.706	0.1117
HA/β-Carotene + ROS	11.38	478,483	668,886	1.398	0.1202
HA/Quercetin + ROS	11.03	1,034,000	1,282,000	1.240	0.1136

## Data Availability

The original contributions presented in this study are included in the article. Further inquiries can be directed to the corresponding author.

## Abbreviations

The following abbreviations are used in this manuscript:

5-LOX	5-Lipoxygenase
15-LOX	15-Lipoxygenase
AANP	N-Succinyl-Ala-Ala-Pro-pNA
AAPH	2,2′-Azobis(2-methylpropionamidine) dihydrochloride
ABTS	2,2′-Azino-bis(3-ethylbenzothiazoline-6-sulfonic acid)
AUC	Area Under the Curve
β-Carotene	Beta-Carotene
CaCl_2_	Calcium chloride
CuCl_2_	Copper (II) chloride
DCFH-DA	2′,7′-Dichlorodihydrofluorescein diacetate
DHA	Docosahexaenoic acid
DPPH	2,2-Diphenyl-1-picrylhydrazyl
ECM	Extracellular Matrix
EGCG	Epigallocatechin gallate
EPA	Eicosapentaenoic acid
FL/NaFluo	Fluorescein (sodium salt)
FRAP	Ferric Reducing Antioxidant Power
GPC	Gel Permeation Chromatography
HA	Hyaluronic Acid
HAases	Hyaluronidases
HASs	Hyaluronan Synthases
HMW HA	High Molecular Weight Hyaluronic Acid
HNE	Human Neutrophil Elastase
HRS	Hydroxyl Radical Scavenging
H_2_O_2_	Hydrogen peroxide
HYBID	Hyaluronan-binding protein involved in hyaluronan depolymerization
IC_50_	Half-maximal inhibitory concentration
LMW HA	Low Molecular Weight Hyaluronic Acid
M_n_	Number-average molecular weight
M_w_	Weight-average molecular weight
OA	Osteoarthritis
ORAC	Oxygen Radical Absorbance Capacity
PBS	Phosphate-Buffered Saline
PEG	Poly (ethylene glycol)
PPE	Porcine Pancreatic Elastase
RI	Refractive Index
RFU	Relative Fluorescence Units
ROS	Reactive Oxygen Species
RvD1	Resolvin D1
RvD2	Resolvin D2
RvDs	Resolvin D series
RvEs	Resolvin E series
SOD	Superoxide Dismutase
SEC	Size Exclusion Chromatography
SPMs	Specialized Pro-resolving lipid Mediators
TBE	Tris-borate–EDTA buffer
TE	Trolox Equivalent
Trolox	(±)-6-Hydroxy-2,5,7,8-tetramethylchromane-2-carboxylic acid
UV-Vis	Ultraviolet–visible spectrophotometry
WST/WST-1	Water-Soluble Tetrazolium Salt
XOD	Xanthine Oxidase
